# A descriptive study of abdominal complications in patients with mild COVID-19 presenting to the emergency department: a single-center experience in Japan during the omicron variant phase

**DOI:** 10.1186/s12876-023-02681-y

**Published:** 2023-02-19

**Authors:** Shuhei Maruyama, Daiki Wada, Takahiro Oishi, Fukuki Saito, Kazuhisa Yoshiya, Yasushi Nakamori, Yasuyuki Kuwagata

**Affiliations:** 1 Department of Emergency and Critical Care Medicine, Kansai Medical University Medical Center, 10-15 Fumizono-cho, Moriguchi, Osaka 570-8507 Japan; 2 Department of Emergency and Critical Care Medicine, Kansai Medical University Hospital, 2-3-1 Shinmachi, Hirakata, Osaka 573-1191 Japan

**Keywords:** COVID-19, Gastrointestinal bleeding, Acute hemorrhagic colitis

## Abstract

**Background:**

COVID-19 is widely known to induce a variety of extrapulmonary manifestations. Gastrointestinal symptoms have been identified as the most common extra-pulmonary manifestations of COVID-19, with an incidence reported to range from 3 to 61%. Although previous reports have addressed abdominal complications with COVID-19, these have not been adequately elucidated for the omicron variant. The aim of our study was to clarify the diagnosis of concomitant abdominal diseases in patients with mild COVID-19 who presented to hospital with abdominal symptoms during the sixth and seventh waves of the pandemic of the omicron variant in Japan.

**Methods:**

This study was a retrospective, single-center, descriptive study. In total, 2291 consecutive patients with COVID-19 who visited the Department of Emergency and Critical Care Medicine, Kansai Medical University Medical Center, Osaka, Japan, between January 2022 and September 2022 were potentially eligible for the study. Patients delivered by ambulance or transferred from other hospitals were not included. We collected and described physical examination results, medical history, laboratory data, computed tomography findings and treatments. Data collected included diagnostic characteristics, abdominal symptoms, extra-abdominal symptoms and complicated diagnosis other than that of COVID-19 for abdominal symptoms.

**Results:**

Abdominal symptoms were present in 183 patients with COVID-19. The number of patients with each abdominal symptom were as follows: nausea and vomiting (86/183, 47%), abdominal pain (63/183, 34%), diarrhea (61/183, 33%), gastrointestinal bleeding (20/183, 11%) and anorexia (6/183, 3.3%). Of these patients, 17 were diagnosed as having acute hemorrhagic colitis, five had drug-induced adverse events, two had retroperitoneal hemorrhage, two had appendicitis, two had choledocholithiasis, two had constipation, and two had anuresis, among others. The localization of acute hemorrhagic colitis was the left-sided colon in all cases.

**Conclusions:**

Our study showed that acute hemorrhagic colitis was characteristic in mild cases of the omicron variant of COVID-19 with gastrointestinal bleeding. When examining patients with mild COVID-19 with gastrointestinal bleeding, the potential for acute hemorrhagic colitis should be kept in mind.

## Background

Severe acute respiratory syndrome coronavirus 2 (SARS-CoV-2) was first identified in December 2019 in Wuhan, China, as the cause of a respiratory illness designated coronavirus disease 2019 (COVID-19). The clinical course ranges from asymptomatic to critically ill, and approximately 5–20% of patients with COVID-19 develop severe pneumonitis, with some progressing to life-threatening respiratory failure, acute respiratory distress syndrome, multiple organ failure and various other pathological conditions [1–5]. The typical clinical symptoms of patients with mild COVID-19 are fever, cough, dyspnea and myalgia or fatigue. Moreover, COVID-19 is widely known to also induce a variety of extrapulmonary manifestations, with gastrointestinal manifestations being the most common of them [6, 7].

The incidence of gastrointestinal symptoms with COVID-19 was reported to range from 3 to 61%. The prevalence of anorexia, nausea/vomiting, diarrhea and abdominal pain with COVID-19 was reported to be 21 to 34.8%, 7 to 26.4%, 9 to 33.7% and 1.9 to 14.5%, respectively [7–10].

In Japan, 1.7 million patients were infected with COVID-19 from the first to the fifth wave (from March 2020 to December 2021). Among the more highly infectious strains, omicron variants BA1, BA2 and BA5 caused widespread infection, and there were up to 20 million patients with COVID-19 during the sixth and seventh waves (from January 2022 to November 2022) in Japan. Although previous reports have addressed abdominal complications with COVID-19, these have not been adequately elucidated for the omicron variant, the latest strain.

As the infection spread in Japan, many patients with mild COVID-19 presented to emergency departments with extra-pulmonary symptoms. Thus, the aim of our study was to clarify the diagnosis of concomitant abdominal diseases in patients with mild COVID-19 who presented to the hospital with abdominal symptoms during the sixth and seventh waves of the pandemic in Japan.

## Methods

### Study design

This study was a retrospective, single-center, descriptive study. In total, 2291 consecutive patients who were diagnosed as having COVID-19 confirmed by polymerase chain reaction or antigen test for SARS-CoV-2 from nasopharyngeal swab samples and who presented to the emergency department of Kansai Medical University Medical Center, Osaka, Japan, between January 1, 2022, and September 30, 2022, were potentially eligible for the study. To target patients with mild COVID-19 and avoid bias, patients delivered by ambulance or who transferred from other hospitals were not included. The inclusion criterion was patients presenting to the emergency department with abdominal manifestations. The exclusion criteria were being pregnant and age under 18 years old. In principle, emergency physicians with 5 to 10 years of experience examined and diagnosed the patients, and blood tests and computed tomography (CT) scans could be performed at any time if necessary. To prevent the transmission of COVID-19, endoscopy cannot be easily performed, and therefore, the performance of endoscopy was not considered mandatory for diagnosis in this study. After application of the exclusion criteria, 183 patients were selected. Clinical outcomes were monitored up to September 30, 2022.

### Data collection

We collected and described physical examination results, medical history, hematological and biochemical data, CT findings and treatments as obtained from the electronic medical records of the patients with SARS-CoV-2 infection. Data collected included sex, age, race, body mass index, abdominal symptoms (nausea and vomiting, abdominal pain, diarrhea, gastrointestinal bleeding and anorexia), extra-abdominal symptoms (cough or sputum, fever, sore throat, headache, fatigue, dysosmia and dysgeusia), days from onset to presentation, origin, vaccination frequency, complicated diagnosis other than that of COVID-19 for abdominal symptoms, comorbidities (hypertension, diabetes mellitus, dyslipidemia, bronchial asthma, chronic obstructive pulmonary disease, cardiovascular disease, chronic renal disease, liver disease, gastrointestinal disease, psychiatric disorder, malignancy disease and autoimmune disease), antiviral treatment received (antiviral drugs and neutralizing antibody therapy) and outcome (outpatient treatment, hospitalization or therapeutic interventions).

### Statistical analysis and ethical concerns

Categorical data are summarized as frequencies and proportion, whereas continuous variables as shown as the median and 25–75th percentile range. Statistical analysis was performed with SPSS 28.0 software (IBM Corp, USA). This study was conducted according to the principles expressed in the Declaration of Helsinki and approved by Kansai Medical University Medical Center—Institutional Review Board (Study Number: 2022193). Due to the retrospective study design, the requirement for written informed consent was waived (Kansai Medical University Medical Center—Institutional Review Board).

## Results

### Patients, manifestations and hospitalizations

**Fig. 1:**
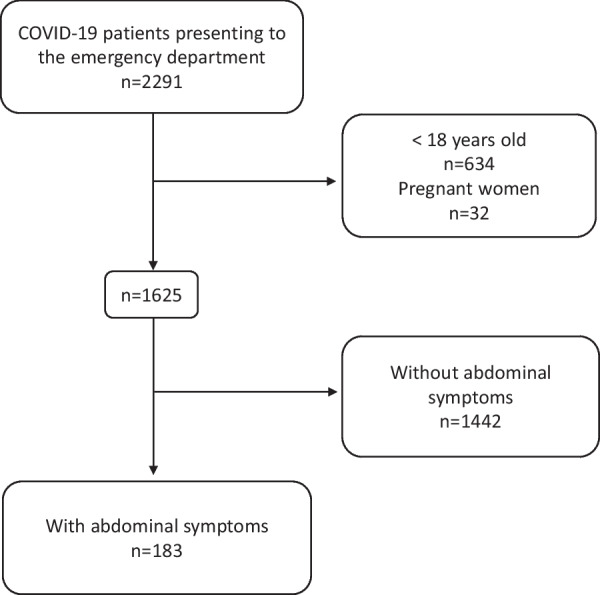
Flow chart of this study

In total, 2291 patients with COVID-19 presented to the emergency department of Kansai Medical University Medical Center, Osaka, Japan, between January and September 2022. After removing the patients meeting the exclusion criteria, 1625 patients were included in the analyses, of whom 183 had abdominal symptoms (Fig. 1). The majority of patients were female (111/183, 61%), and the median age was 42 (range 18–90) years old. The number of patients with each abdominal symptom was as follows: nausea and vomiting (86/183, 47%), abdominal pain (63/183, 34%), diarrhea (61/183, 33%), gastrointestinal bleeding (20/183, 11%) and anorexia (6/183, 3.3%). Extra-abdominal symptoms included the following: cough or sputum (90/183, 49%), fever (78/183, 43%), sore throat (65/183, 36%), headache (43/183, 23%), fatigue (33/183, 18%), dysgeusia (4/183, 2.2%) and dysosmia (1/183, 0.5%). Nearly one half of the patients (89/183, 49%) underwent chest and abdominal CT, whereas one third (58/183, 32%) underwent only chest CT. Most patients (146/183, 80%) presented directly, and the others (37/183, 20%) were referred from public health centers or clinics. Only some patients (28/183, 15%) were hospitalized for treatment (Table 1).

**Table 1:** Baseline characteristics, clinical presentation, CT scanning and treatment of patients with abdominal manifestations

Characteristics	Total N = 183
Male, *N*	72 (39%)
Age (years)	42 [30–55]
Asian, *N*	183 (100%)
Body mass index (kg/m^2^)	22 [20–25]
Comorbidities, *N*	
Hypertension	14 (7.7%)
Diabetes mellitus	13 (7.1%)
Dyslipidemia	8 (4.4%)
Bronchial asthma	14 (7.7%)
COPD	1 (0.5%)
Cardiovascular disease	6 (3.3%)
CKD	4 (2.2%)
Liver disease	3 (1.6%)
Gastrointestinal disease	9 (4.9%)
Psychiatric disorder	19 (10.4%)
Malignancy disease	10 (5.5%)
Autoimmune disease	8 (4.4%)
Abdominal manifestations, *N*	
Nausea and vomiting	86 (47%)
Abdominal pain	63 (34%)
Diarrhea	61 (33%)
Gastrointestinal bleeding	20 (11%)
Anorexia	6 (3.3%)
Extra-abdominal manifestations, *N*	
Cough or sputum	90 (49%)
Fever	78 (43%)
Sore throat	65 (36%)
Headache	43 (23%)
Fatigue	33 (18%)
Dysgeusia	4 (2.2%)
Dysosmia	1 (0.5%)
Vaccination frequency, *N*	
< 2	58 (32%)
2	58 (32%)
3	58 (32%)
4	5 (2.2%)
Missing data	4 (2.2%)
CT scan, *N*	
None	36 (20%)
Chest	58 (32%)
Chest and abdominal	89 (49%)
Treatment, *N*	
Nirmatrelvir/ritonavir	9 (4.9%)
Remdesivir	26 (14%)
Molnupiravir	16 (8.7%)
Sotrovimab	15 (8.2%)
Casirivimab and imdevimab	17 (9.3%)
Steroid	2 (1.1%)
Onset to presentation (days)	4 [2–6]
Origin, *N*	
None	146 (80%)
Public health center	33 (18%)
Clinic	4 (2.2%)
Hospital	0 (0%)
Hospitalization, *N*	
Yes	28 (15%)
No	155 (85%)

Data are expressed as *N* (%) or median [1st IQR-3rd IQR]

*CT* Computed tomography, *COPD* Chronic obstructive pulmonary disease, *CKD* Chronic kidney disease

### Diagnosis

**Fig. 2:**
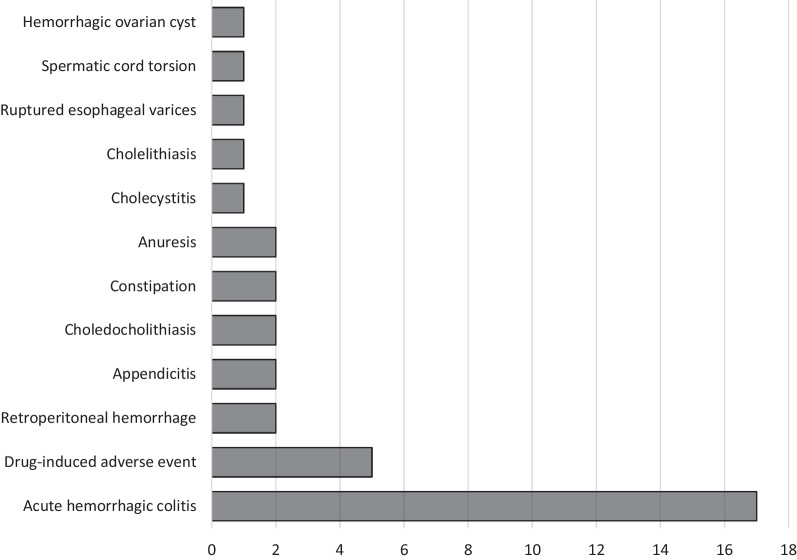
Number of patients per diagnosis of abdominal manifestations complicating COVID-19

Most patients (146/183, 80%) did not have a complicated diagnosis other than that of COVID-19 for abdominal symptoms. Among the remaining 37 patients, 17 were diagnosed as having acute hemorrhagic colitis, five had drug-induced adverse events, two had retroperitoneal hemorrhage, two had appendicitis, two had choledocholithiasis, two had constipation, two had anuresis and one patient each had cholecystitis, cholelithiasis, ruptured esophageal varices, spermatic cord torsion and hemorrhagic ovarian cyst, respectively. Pharmaceutical therapy suspected of causing drug-related complications included nirmatrelvir/ritonavir and molnupiravir in two patients each and casirivimab and imdevimab in one patient each (Fig. 2) (Table 2).

**Table 2:** Summary of patients with abdominal manifestations diagnosed as other than COVID-19

No	Diagnosis	Sex	Age	BMI	Abdominal clinical presentation	Comorbidities	Vaccination frequency	D-dimer (μg/mL)	CRP (mg/dL)	Anti-S antibody (U/mL)	CT scan	Pneumonia	Onset to presentation (days)	Origin	ED/hospitalization	Treatment
1	Acute hemorrhagic colitis (descending)	M	23	24	Abdominal pain Gastrointestinal bleeding	Pneumonitis	2	1.7	1.3	559	Chest Abdominal	No	3	Clinic	Hospitalization	Conservation
2	Acute hemorrhagic colitis (sigmoid)	M	23	24	Abdominal pain Gastrointestinal bleeding Nausea	None	2	< 0.2	2.5	515	Chest Abdominal	No	5	PHC	ED	Conservation
3	Acute hemorrhagic colitis (splenic flexure to sigmoid)	M	23	17	Abdominal pain Gastrointestinal bleeding Diarrhea, Vomiting	None	0	1.3	0.8	< 0.4	Chest Abdominal	No	5	None	ED	Conservation
4	Acute hemorrhagic colitis (sigmoid)	M	27	24	Gastrointestinal bleeding	None	2	< 0.2	0.5	880	Chest Abdominal	No	5	PHC	ED	Conservation
5	Acute hemorrhagic colitis (descending)	M	30	24	Abdominal pain Gastrointestinal bleeding	None	2	0.4	0.7	1243	Chest Abdominal	No	7	None	ED	Conservation
6	Acute hemorrhagic colitis (descending to sigmoid)	F	34	19	Abdominal pain Gastrointestinal bleeding	None	2	0.3	0.4	6685	Chest Abdominal	No	6	None	ED	Conservation
7	Acute hemorrhagic colitis (splenic flexure to descending)	M	39	25	Gastrointestinal bleeding	None	0	0.6	4.3	< 0.4	Chest Abdominal	No	1	None	ED	Conservation
8	Acute hemorrhagic colitis (descending)	F	42	23	Abdominal pain Gastrointestinal bleeding Diarrhea	None	2	0.4	0.9	< 0.4	Chest Abdominal	No	3	None	ED	Conservation
9	Acute hemorrhagic colitis (descending)	F	42	Missing	Abdominal pain Gastrointestinal bleeding	Kikuchi disease	3	< 0.2	3.7	18,981	Chest Abdominal	No	2	None	ED	Conservation
10	Acute hemorrhagic colitis (descending to sigmoid)	F	44	29	Abdominal pain Gastrointestinal bleeding	HT Diverticulum hemorrhage	2	0.9	0.1	1109	Chest Abdominal	No	3	Clinic	ED	Conservation
11	Acute hemorrhagic colitis (descending to sigmoid)	F	45	22	Abdominal pain Gastrointestinal bleeding	Arrhythmia	0	0.9	0.1	< 0.4	Chest Abdominal	No	3	None	Hospitalization	Conservation
12	Acute hemorrhagic colitis (splenic flexure to sigmoid)	F	52	18	Abdominal pain Gastrointestinal bleeding	Ischemic colitis Uterine fibroid	3	3.8	0.3	52,443	Chest Abdominal	No	7	None	ED	Conservation
13	Acute hemorrhagic colitis (descending)	F	59	24	Abdominal pain Gastrointestinal bleeding	Uterine fibroid	0	0.5	1.7	< 0.4	Chest Abdominal	No	3	None	ED	Conservation
14	Acute hemorrhagic colitis (splenic flexure to descending)	F	61	20	Abdominal pain Gastrointestinal bleeding	None	4	0.4	0.1	96,338	Chest Abdominal	No	4	None	ED	Conservation
15	Acute hemorrhagic colitis (descending)	F	65	23	Abdominal pain Gastrointestinal bleeding	Rheumatoid arthritis	3	< 0.2	0.1	1448	Chest Abdominal	No	2	PHC	Hospitalization	Conservation
16	Acute hemorrhagic colitis (splenic flexure to sigmoid)	F	66	24	Abdominal pain Gastrointestinal bleeding	Pulmonary tuberculosis	3	1.2	6.1	5524	Chest Abdominal	No	8	PHC	Hospitalization	Conservation
17	Acute hemorrhagic colitis (descending)	F	84	20	Abdominal pain Gastrointestinal bleeding	GERD Endometriosis	2	0.9	4.5	38,839	Chest Abdominal	Yes	6	None	Hospitalization	Conservation
18	Drug-induced adverse event (Casirivimab and imdevimab)	M	47	34	Nausea	HT, HL, angina pectoris	2	0.8	0.3	633	Chest Abdominal	No	5	None	Hospitalization	Conservation
19	Drug-induced adverse event (nirmatrelvir/ritonavir)	F	49	19	Abdominal pain Nausea	None	3	0.2	1	2809	Chest	Yes	10	None	ED	Conservation
20	Drug-induced adverse event (molnupiravir)	F	66	28	Abdominal pain	BA, DM, HT, Arrhythmia	3	0.8	0.2	498	Chest	Yes	5	None	ED	Conservation
21	Drug-induced adverse event (nirmatrelvir/ritonavir)	M	67	Missing	Nausea and vomiting	None	3	0.4	1.4	5641	Chest Abdominal	No	5	None	ED	Conservation
22	Drug-induced adverse event (molnupiravir)	F	71	Missing	Diarrhea	Auto immune hepatitis	0	1.1	1.1	< 0.4	Chest	Yes	3	None	ED	Conservation
23	Retroperitoneal hemorrhage	M	41	28	Abdominal and back pain	HL HU	3	1.1	6.2	6790	Chest Abdominal	No	5	None	Hospitalization	Embolization
24	Retroperitoneal hemorrhage	M	56	22	Abdominal pain	None	3	1.3	2.1	10,553	Chest Abdominal	No	1	None	Hospitalization	Embolization
25	Appendicitis	F	53	20	Abdominal pain Diarrhea	None	3	0.5	16.9	5466	Chest Abdominal	No	5	None	Hospitalization	Surgery
26	Appendicitis	M	75	22	Abdominal pain	Duodenal ulcer	3	2.3	14	3693	Chest Abdominal	No	3	PHC	Hospitalization	Conservation
27	Choledocholithiasis	F	24	40	Abdominal pain	None	2	9.9	0.4	9577	Chest Abdominal	No	8	None	Hospitalization	Endoscopy
28	Choledocholithiasis	F	31	25	Abdominal pain	None	3	1.8	0.5	N/A	Chest Abdominal	No	9	None	Hospitalization	Endoscopy
29	Constipation	F	42	23	Abdominal pain	None	3	N/A	N/A	N/A	Chest Abdominal	No	8	None	ED	Conservation
30	Constipation	F	81	Missing	Constipation	COPD Dyslipidemia	3	1.1	0.9	18,461	Chest	No	4	PHC	ED	Conservation
31	Anuresis	M	74	21	Anuresis	Appendicitis Prostatitis	4	N/A	N/A	N/A	Chest Abdominal	No	7	None	ED	Conservation
32	Anuresis	F	90	18	Abdominal pain	HT, gastric ulcer Pneumonitis, pyelonephritis	2	2.5	0.3	97.5	Chest Abdominal	Yes	4	None	ED	Conservation
33	Cholecystitis	M	55	26	Abdominal pain	HT	3	2.0	28.1	15,422	Chest Abdominal	No	6	None	Hospitalization	Surgery
34	Cholelithiasis	F	44	24	Abdominal pain	None	3	0.4	2.5	54,505	Chest Abdominal	No	2	None	ED	Conservation
35	Ruptured esophageal varices	M	74	22	Gastrointestinal bleeding	DM, HT, LC	0	1.1	1.5	< 0.4	Chest Abdominal	Yes	4	PHC	Hospitalization	Endoscopy
36	Spermatic cord torsion	M	21	21	Abdominal pain Orchialgia	None	0	< 0.2	0.7	< 0.4	None	N/A	8	PHC	Hospitalization	Surgery
37	Hemorrhagic ovarian cyst	F	22	21	Abdominal pain	None	3	< 0.2	0.1	99,004	Chest Abdominal	No	1	Clinic	ED	Conservation

*BMI* Body mass index, *CRP* C-reactive protein, *CT* Computed tomography, *ED* Emergency department, *PHC* Public health center, *HT* Hypertension, *HL* Hyperlipidemia, *GERD* Gastroesophageal reflux disease, *BA* Bronchial asthma, *DM* Diabetes mellitus, *HU* Hyperuricemia, *COPD* Chronic obstructive pulmonary disease, *LC* Liver cirrhosis

### Characteristics of the 17 patients diagnosed as having acute hemorrhagic colitis

The majority of patients were female (11/17, 65%), and median age was 42 (range 23–84) years old. The number of patients with each abdominal symptom was as follows: gastrointestinal bleeding (17/17, 100%) and abdominal pain (15/17, 88%), diarrhea (2/17, 12%), nausea (1/17, 6%) and vomiting (1/17, 6%). The laboratory data were as follows: D-dimer (µg/mL), CRP (mg/dL) and anti-SARS-CoV-2 S antibody (U/mL) (0.5 [0.2–0.9], 0.8 [0.3–2.5] and 1109 [0–6685], median [1st IQR–3rd IQR], respectively). The CT findings of all patients showed the colon appearing as thickening along with peri-colic fat stranding (descending 7/17, descending to sigmoid 3/17, splenic flexure to sigmoid 3/17, sigmoid 2/17 and splenic flexure to descending colon 2/17).

## Discussion

We described patients with mild COVID-19 and abdominal symptoms who presented to our emergency department during the sixth and seventh waves (from January 2022 to September 2022) in which the SARS-CoV-2 omicron variants BA1, BA2 and BA5 were widespread. About one tenth of the patients (183/1625, 11%) experienced some abdominal symptoms. In 37 patients, diagnoses other than COVID-19 as the cause of abdominal symptoms included acute hemorrhagic colitis, drug-related adverse events, retroperitoneal bleeding, appendicitis, cholangitis, constipation and urinary retention in that order.

**Fig. 3:**
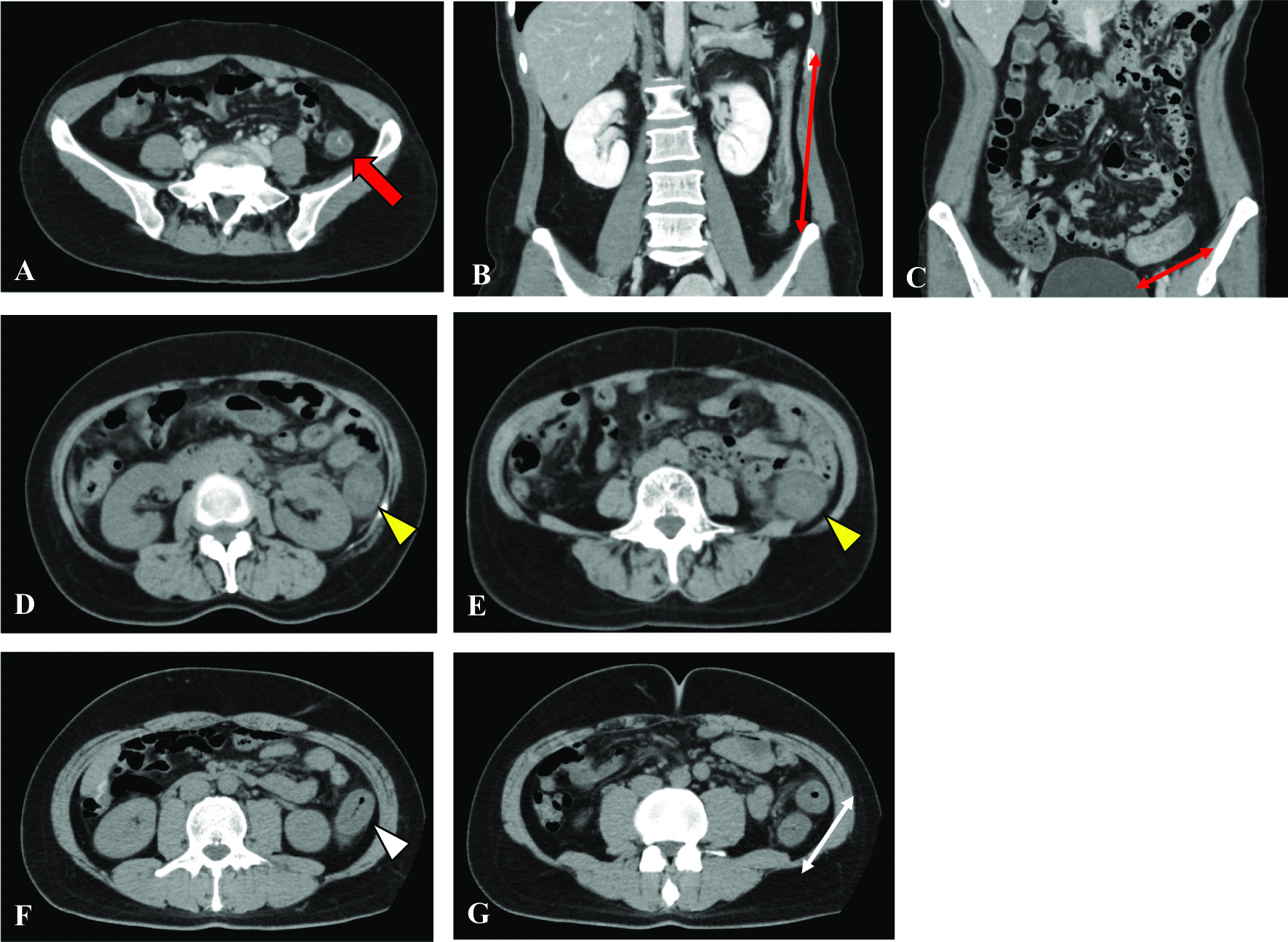
Representative computed tomography (CT) images from patients with COVID-19 and acute hemorrhagic colitis. **A**–**C** Axial and coronal CT images of a 45-year-old patient showing evidence of acute hemorrhagic colitis involving the descending to sigmoid colon appearing as thickening along with peri-colic fat stranding (red arrow and thin red arrows). **D**, **E** Axial CT images of a 59-year-old patient revealed acute hemorrhagic colitis involving the descending colon that showed marked thickening with peri-colic fat stranding (yellow arrowheads). **F**, **G** Axial CT images of a 39-year-old patient revealed acute hemorrhagic colitis involving the splenic flexure to descending colon that showed marked thickening with peri-colic fat stranding (white arrowhead and thin white double-headed arrow)

Among the 20 patients with COVID-19 who had gastrointestinal bleeding, 85% (17/20) were diagnosed as having acute hemorrhagic colitis, which was characteristic in this study (Fig. 3). The diagnostic criteria of acute hemorrhagic colitis were satisfied by two elements, the colon appearing as thickening along with peri-colic fat stranding on abdominal CT, and gastrointestinal bleeding as a symptom. All colitis was localized in the left hemi-colon, from the splenic flexure to the sigmoid colon. The lesion site was similar to that of ischemic colitis, so it was necessary to determine a differential diagnosis by imaging. A previous study reported that ischemic colitis was more common in non-COVID-19 women over the age of 49 years [11, 12]. However, 11/17 patients diagnosed as having acute hemorrhagic colitis were younger than 50 years in the present study. Ischemic colitis is commonly categorized into two classical patterns, occlusive and nonocclusive. However, there was little elevation of D-dimer (< 3.8 µg/mL) suggesting thrombosis in the patients with acute hemorrhagic colitis in this study. Although no endoscopic or pathological examination was performed, the epidemiology was thought to differ from that of ischemic colitis. COVID-19 is known to be a systemic disease, with a specific tropism for endothelial cells that leads to microvascular disease with multisystemic involvement, and therefore, it also affects the gastrointestinal system [13]. Bleeding and ischemic manifestations are also frequent, with spontaneous hematomas in soft tissues being the most common. Ischemic and hemorrhagic abdominal complications such as ischemic colitis, small bowel ischemia, retroperitoneal bleeding and others may occur in patients with COVID-19 [14–19]. The overall rate of gastrointestinal bleeding in patients with COVID-19 reportedly ranged from 1.1 to 13%, with most patients with gastrointestinal bleeding being critically ill men with a mean age of 67.5 years [14]. COVID-19-induced colitis that presents with abdominal pain, watery diarrhea and gastrointestinal bleeding consistent with an acute hemorrhagic colitis was reported as an uncommon occurrence [20]. Two injury mechanisms of inflammatory responses induced by COVID-19 have been reported: one is mediated by angiotensin-converting-enzyme (ACE)-2 receptors, and the other is independent of ACE-2 receptors [21–23]. Furthermore, as ACE-2 receptors are widely expressed not only in the airway and alveolar epithelial cells but also in the intestinal epithelial cells, renal epithelial cells, myocardial cells and vascular endothelial cells, SARS-CoV-2 infection induces systemic local inflammation via systemic ACE-2 receptors [21–24]. The independent mechanism is the accumulation of inflammatory cytokines such as IL-6, IL-7, TNF and inflammatory chemokines, which cause an overwhelming viremic response with resultant injury to the digestive mucosa that damages the digestive system through a viral inflammatory response [25–29].

**Fig. 4:**
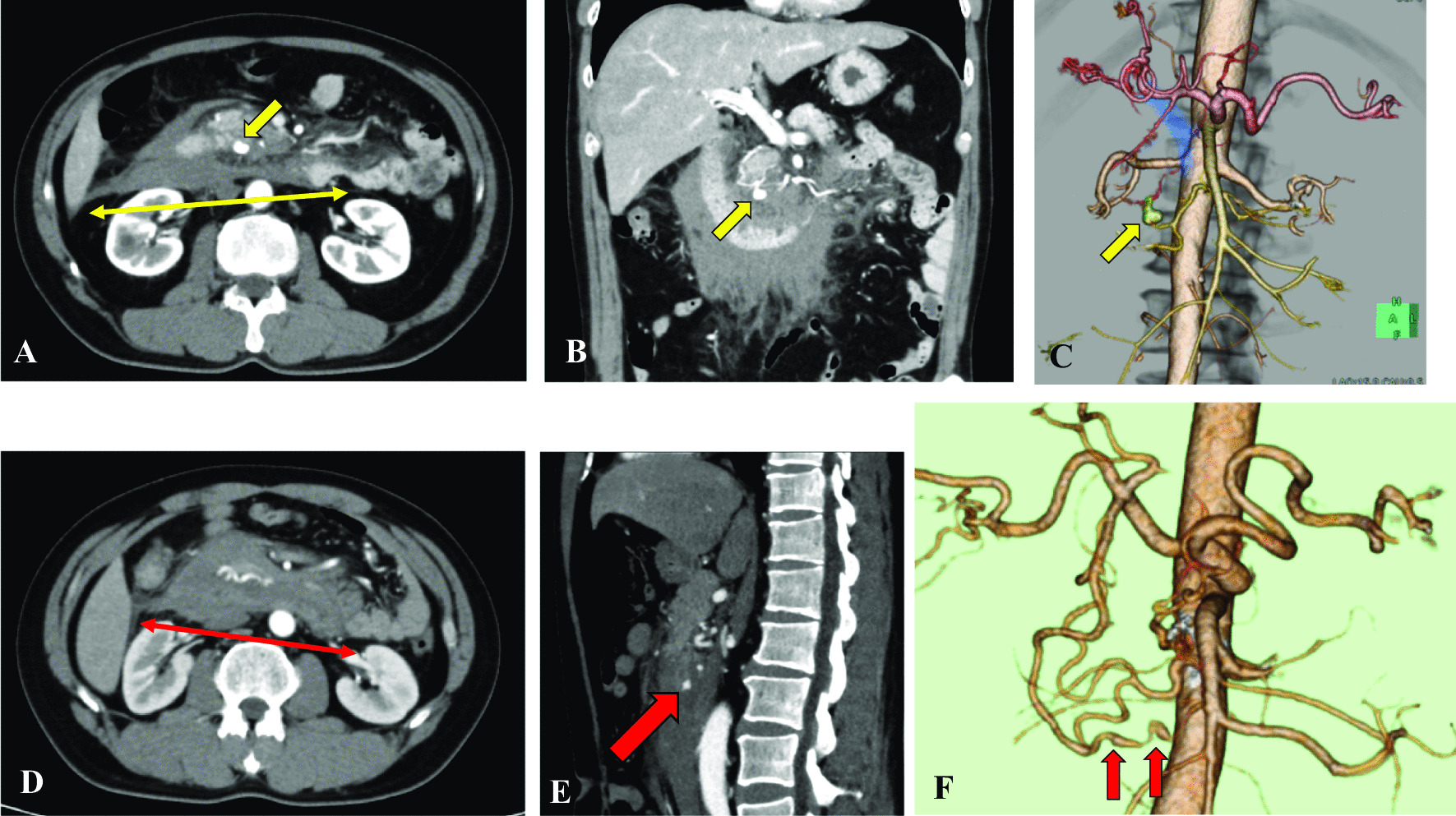
Representative computed tomography (CT) images of retroperitoneal hemorrhage in patients with COVID-19. **A**–**C** Axial, coronal and 3D CT images from a 56-year-old patient show evidence of retroperitoneal hemorrhage (thin yellow double-headed arrow) with pseudoaneurysm of the inferior pancreaticoduodenal artery (yellow arrows). **D**–**F** Axial, sagittal and 3D CT images from a 41-year-old patient show evidence of retroperitoneal hemorrhage (thin red double-headed arrow, **D**) with pseudoaneurysm of the anterior inferior pancreaticoduodenal artery (red arrows)

There were two patients in the present study with retroperitoneal hemorrhage due to a ruptured visceral artery aneurysm (Fig. 4), which is a relatively rare condition. Its reported prevalence is approximately 1% in the total population, and it is found in 0.01–0.2% of autopsy cases, most of which are detected following rupture [30, 31]. Although the increased risk of bleeding could be related to endothelial dysfunction, coagulopathy or disseminated intravascular coagulation in COVID-19, the risk of bleeding in patients with mild COVID-19 was not reported to increase [32]. There have been some reports of retroperitoneal hemorrhage in severely or critically ill patients with COVID-19 requiring anticoagulant therapy [13, 33], but the COVID-19 in the two patients in our study was mild with no evidence of pneumonitis, and neither patient received antithrombotic therapy. Although the relation of retroperitoneal hemorrhage with mild COVID-19 remains unclear, our two patients required emergency intervention due to lethal complications.

This study has several limitations. First, as it was a retrospective, single-center, observational, descriptive study, there was possible bias in relation to the patient population. Second, we diagnosed acute hemorrhagic colitis based on symptoms and CT scans. No endoscopy or histological or pathogenic examination was performed. Therefore, other diseases, such as bacterial and viral diseases, could not be adequately ruled out.

## Conclusion

Our study showed that acute hemorrhagic colitis was characteristic in patients with mild COVID-19 of the omicron variant who had gastrointestinal bleeding. About 10% of these patients with abdominal symptoms had acute hemorrhagic colitis, and thus, its occurrence might be relatively more frequent than previously reported. When examining patients with mild COVID-19 and gastrointestinal bleeding, acute hemorrhagic colitis should be kept in mind. About 1% of the patients with abdominal symptoms had retroperitoneal hemorrhage, so even patients with mild disease might suffer retroperitoneal hemorrhage induced by COVID-19.

## Data Availability

The datasets analyzed during the current study are available from the corresponding author on reasonable request.

## Abbreviations

COVID-19: Coronavirus disease 2019
CT: Computed tomography
ACE: Angiotensin-converting-enzyme
